# Myocardial T2 mapping reveals age- and sex-related differences in volunteers

**DOI:** 10.1186/s12968-015-0118-0

**Published:** 2015-02-06

**Authors:** Florian Bönner, Niko Janzarik, Christoph Jacoby, Maximilian Spieker, Bernhard Schnackenburg, Felix Range, Britta Butzbach, Sebastian Haberkorn, Ralf Westenfeld, Mirja Neizel-Wittke, Ulrich Flögel, Malte Kelm

**Affiliations:** Department of Cardiology, Pulmonology and Vascular Medicine, Heinrich Heine University, Medical Faculty, Moorenstraße 5, 40225 Düsseldorf, Germany; Department of Molecular Cardiology, Heinrich Heine University, Medical Faculty, Düsseldorf, Germany; Philips Healthcare, Hamburg, Germany; CARID (Cardiovascular Research Institute Düsseldorf), Düsseldorf, Germany

**Keywords:** Cardiovascular magnetic resonance, T2 mapping, Volunteer study, SENC

## Abstract

**Background:**

T2 mapping indicates to be a sensitive method for detection of tissue oedema hidden beyond the detection limits of T2-weighted Cardiovascular Magnetic Resonance (CMR). However, due to variability of baseline T2 values in volunteers, reference values need to be defined. Therefore, the aim of the study was to investigate the effects of age and sex on quantitative T2 mapping with a turbo gradient-spin-echo (GRASE) sequence at 1.5 T. For that reason, we studied sensitivity issues as well as technical and biological effects on GRASE-derived myocardial T2 maps. Furthermore, intra- and interobserver variability were calculated using data from a large volunteer group.

**Methods:**

GRASE-derived multiecho images were analysed using dedicated software. After sequence optimization, validation and sensitivity measurements were performed in muscle phantoms *ex vivo* and *in vivo*. The optimized parameters were used to analyse CMR images of 74 volunteers of mixed sex and a wide range of age with typical prevalence of hypertension and diabetes. Myocardial T2 values were analysed globally and according to the 17 segment model. Strain-encoded (SENC) imaging was additionally performed to investigate possible effects of myocardial strain on global or segmental T2 values.

**Results:**

*Ex vivo* studies in muscle phantoms showed, that GRASE-derived T2 values were comparable to those acquired by a standard multiecho spinecho sequence but faster by a factor of 6. Besides that, T2 values reflected tissue water content. The *in vivo* measurements in volunteers revealed intra- and interobserver correlations with R^2^=0.91 and R^2^=0.94 as well as a coefficients of variation of 2.4% and 2.2%, respectively. While global T2 time significantly decreased towards the heart basis, female volunteers had significant higher T2 time irrespective of myocardial region. We found no correlation of segmental T2 values with maximal systolic, diastolic strain or heart rate. Interestingly, volunteers´ age was significantly correlated to T2 time while that was not the case for other coincident cardiovascular risk factors.

**Conclusion:**

GRASE-derived T2 maps are highly reproducible. However, female sex and aging with typical prevalence of hypertension and diabetes were accompanied by increased myocardial T2 values. Thus, sex and age must be considered as influence factors when using GRASE in a diagnostic manner.

**Electronic supplementary material:**

The online version of this article (doi:10.1186/s12968-015-0118-0) contains supplementary material, which is available to authorized users.

## Background

One of the major advantages of Cardiovascular Magnetic Resonance (CMR) in comparison to other non-invasive imaging modalities represents its superior soft tissue contrast. This contrast is determined by myocardial T1 and T2 relaxation properties which can be used for imaging myocardial diseases without the need of additional contrast enhancement [1,2].

T2-weighted CMR has proven to be useful in discriminating acute from chronic myocardial infarct lesions according to the presence or absence of oedema [3]. However, T2-weighted sequences have a number of disadvantages: T2 sequences are prone to artifacts and have low sensitivity, making the image interpretation difficult [4]. On the other hand, using the appropriate sequence, T1 and T2 relaxation times can directly be derived from the images, and thus the relaxation values can be investigated with greater objectivity than a threshold-based grading of myocardial signal intensity [5]. While native T1 mapping has shown to more likely display increased extracellular space or fibrosis [6], T2 relaxation time was reported to be a quite sensitive marker of myocardial oedema after cardiac ischemia and reperfusion [1,2,7].

Several approaches to quantify myocardial T2 relaxation in a global manner have been set up in patients with ubiquitous myocardial diseases like familiar hypertrophy or Morbus Fabry [8-10]. Recently, localized T2 mapping on the basis of gradient echo sequences with preceding T2 preparation (0, 24, 55 ms) have been investigated in volunteers as well as patients with suspected focal edema [11-15]. However, as a result of different hardware or sequence parameters in use for myocardial T2 mapping, reference values of volunteers may considerably differ. Moreover, the biological heterogeneity of myocardial texture in hearts of volunteers and patients as a result of age and sex might have a significant impact on myocardial T2 reference values [16,17]. Interestingly, recent T2 mapping approaches have indicated that local myocardial wall motion and heart rate might have influence on local myocardial T2 values [14]. Thus, myocardial T2 values should be obtained in a large group of non-diseased controls for reference purposes in each T2 mapping sequence.

Against this background, the present study evaluates a turbo gradient- and spin-echo (GRASE) sequence for quantification of local myocardial T2 values at 1.5 Tesla. To this end, 74 volunteers of different sex and a wide range of age were enrolled. We hypothesize that age and sex have an impact on myocardial T2 values.

## Methods

The ethical board of Heinrich Heine University Düsseldorf approved the present study (application number 4307). All participants were enrolled after informed consent was obtained. The study complies with the declaration of Helsinki.

### Volunteers

In total 74 volunteers were enrolled for the study. Depending on their age, two groups were formed (<35 years; younger volunteers, >35 years; older volunteers) and compared in Table 1. Volunteers were characterized by lacking of any cardiovascular disease, no symptoms of inflammation, prohibition of alcohol intake >48 h, absence of hereditary cardiovascular diseases, and normal electrocardiogram. Additionally, older volunteers without a medical history of coronary artery disease were seen in our regular ambulance. They received regular functional CMR including stress testing and Late Gadolinium Enhancement (LGE). In the present study we included volunteers without structural myocardial disease as delineated by LGE and without perfusion defects indicating a relevant coronary artery disease as identified by perfusion imaging and without atrial fibrillation. CMR findings were analysed in a blinded fashion by two experienced observers. The group of older volunteers showed a typical appearance of cardiovascular risk factors like hypertension and diabetes (Table 1) which were not defined as exclusion criteria.

**Table 1:** **Volunteer characteristics**

**Characteristics of volunteer cohort (n = 74)**			
	**Young (n = 40)**	**Old (n = 34)**	**p-value**
Age (Y)	27.5 ± 7.4	63.8 ± 9.9	<0.01
Female	35.6%	39%	n.s.
Cardiovascular risk factors			
Hyoertension	2.6%	63.6%	<0.01
Diabetes	0%	12%	<0.01
Hyperlipidemia	5.1%	39.4%	<0.01
Current smokers	20.5%	15.6%	<0.05
Presentation characteristics			
Recent infection	0%	0%	n.s.
TnT (pg/ml)	<14	<14	n.s.
ECG abnormalities	0%	0%	n.s.
NYHA class	n.a.	n.a.	n.s.
cMRI parameters			
Myocardial weight index (Heart mass/body surface area; g/m^2^)	62.2 ± 16.4	61.9 ± 17.2	n.s.
Ejection fraction (%)	62.8 ± 11.4	66.7 ± 10.2	n.s.
LGE	no	no	n.s.
T2w MRI	no	no	n.s.

### CMR

Phantom measurements as well as volunteer scans were performed using a 1.5 T scanner (Archieva, Philips, Best Netherlands) with a 32-channel phased array coil.

For T2 mapping we applied the GRASE sequence which has already been used for quantitative T2 measurement in liver and brain [18-20]. This sequence combines the TSE and echo-planar imaging methods by using a train of refocusing 180° pulses and an odd number of additional gradient echoes for each spin echo. This sequence was used with cardiac triggering and respiration navigator gating with the following parameters: TR = 1 RR interval, number of echo images = 15, echo spacing 10 ms, leading to an echo train of 150 ms, number of gradient echoes for segmented acquisition = 3 (EPI factor), FA = 90°, spatial resolution: 2 × 2 × 10 mm^3^, parallel imaging (SENSE) with an acceleration factor of 2, k-space data acquired with cartesian encoding scheme. For blood saturation a double inversion (black-blood) pulse was used.

### Phantom experiments

Detailed information about the phantom experiments can be found in the Additional file 1. We validated GRASE-derived T2 values with a conventional multi echo spin echo (MESE) sequence in commercially available cow meat (Additional file 1: Figure S1) in order to find possible effects of EPI-like segmentation on T2.

Besides validation purposes, the phantom study was set up to test the sensitivity of myocardial T2 quantification in terms of focal and global tissue water changes. In the first experiment, cow muscle (M. erector spinae) was cut into 15 small pieces of 20 g and 14 pieces were dehydrated by vacuum concentration (SpeedVac, Thermo Fisher Scientific) at given durations (0.5, 1, 2, 3, 4, 5, 6, 7, 8, 10, 11, 15, 20, 24 h). All pieces of meat were imaged at once with GRASE. The percentage of tissue water was calculated as follows: the difference of wet weight to dry weight was divided by wet weight. This ratio (0.63) was constant between 20 and 24 h, indicating complete tissue drying and the relative tissue water mass of the meat to be 63%. This value was set to 100% of tissue water.

### Volunteer scans

The measurement protocol was conducted in the following order: scout and reference scans, cine-loops in 3 short axis (SAX) slices and in four chamber view (4CH) (balanced SSFP cine-MRI: TR/TE = 2.9/1.5 ms, FA = 60°, res = 8 × 1.5 × 1.5 mm^3^, 35 phases, breath-hold), T2-weighted imaging in the respective SAX (TSE-STIR: TR = 2 RR-intervals, TE = 58 ms, spatial resolution = 2 × 2 × 10 mm^3^, imaging in mid-diastole, breath-hold), GRASE in the respective 3 SAX (GRASE: TR = 1 cardiac cycle, EPI factor 3, 15 echos with 10 ms inter echo spacing, FA = 90°, resolution = 2 × 2 × 10 mm^3^, end-diastolic trigger), and strain-encoded imaging in two chamber view (2CH), 4CH and the respective SAX (SENC imaging: TR = 25 ms, TE = 0.9 ms, FA = 30°, spatial resolution: 2.5 × 2.5 × 10 mm^3^). Older volunteers (>35 years), enrolled in our regular ambulance, additionally received Gadolinium DTPA (Gadovist, Bayer Healthcare) for myocardial stress-testing and LGE. The latter was performed using a 3-dimensional gradient -spoiled turbo fast field echo sequence with an nonselective 180° inversion-recovery pre-pulse-triggered to end-diastole which covered the whole ventricle (TR/TE = 3.2/1.16 ms; FA = 15°; spatial resolution: 1.5 × 1.7 × 10 mm^3^, patient-adapted prepulse delay = 200–300 ms, breath-hold).

### T2 mapping post processing

**Figure 1:**
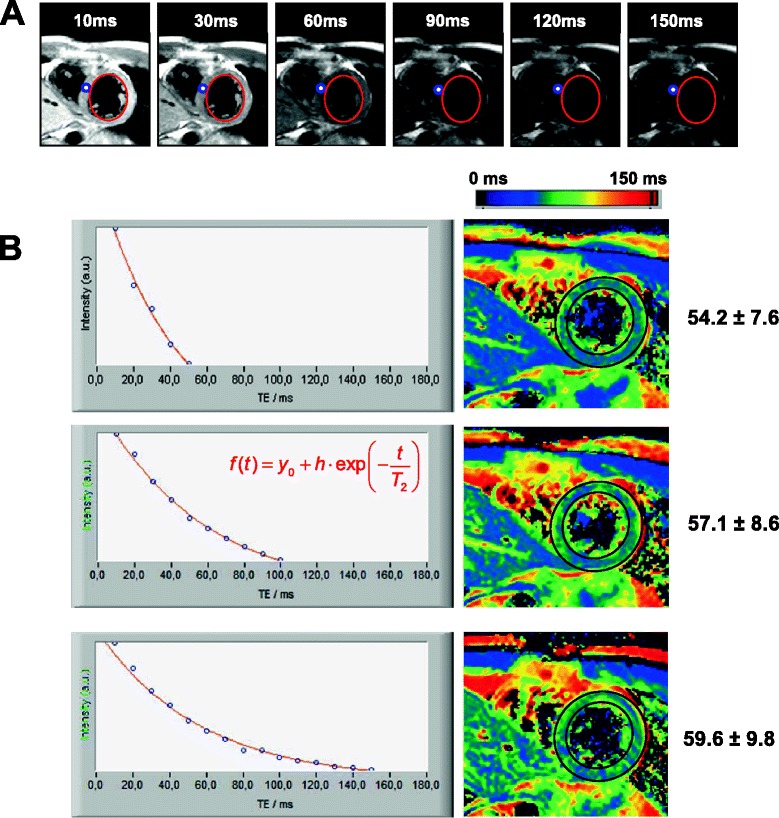
**Principle of T2 map calculation. (A)** Example of 6 GRASE-derived echo images with echo times given in the figures. Note, there is only very little cardiac motion between 10 and 150 ms. **(B)** An exponential fit was performed for each image pixel (exemplarily for the blue circle in the anterior wall of A) with amplitude (h) and damping (1/T_2_) as fit parameters (fixed bias, y_0_). T2 value calculation in myocardial tissue was performed for 5, 10 and 15 echoes with 10 ms interecho time. T2 maps are illustrated by a colour-coded map, assigning 0 ms to black and 150 ms to red.

T2 maps of the respective object (phantom or volunteer) were generated off-line using software based on the graphical programming language LabVIEW (National Instruments, Austin, TX). An exponential decay curve was fitted to the intensity decline of each pixel within the images obtained from the multi echo sequence (Figure 1A). The bias was calculated from the noise of all echo images and assumed to be constant, so that the problem could be linearized and the regression coefficient (R^2^) could be used as a goodness-of-fit parameter in order to exclude accidental values. If R^2^ was not within a tolerance interval chosen to be 0.7−1, the corresponding T_2_ value was not considered for further calculations. The resulting T_2_ constants were colour-coded using the spectral look-up table (Figure 1B).

### Data analysis for T2 quantification and strain encoded imaging

Endo- and epicardial contours were manually drawn in reference images and automatically overlayed to the T2 map. Segmentation was done off-line using the same software as for the generation of T2 maps [7]. For this, a starting point was chosen at the posterior insertion of the right ventricle. Depending on the SAX location, 4 or 6 segments were generated automatically in a clockwise manner according to the 17 segment model of the AHA omitting segment 17 [21]. The contours were drawn by two independent observers omitting “slow flow” ventricular blood or epicardial fat. The coefficient of variation (CoV) was calculated as the ratio of the standard deviation of the inter- or intraobserver differences divided by the mean of the measurement. Time interval between intraobserver analysis was 28 ± 5 days.

To evaluate the role of wall movement on T2 values within the same segment, we acquired myocardial strain values by strain encoded imaging (SENC) of each myocardial segment analysed. Strains were derived from SENC images using dedicated software (Diagnosoft MAIN, version 1.06, Diagnosoft Inc., Palo Alto, California). Circumferential strain was calculated for 12 myocardial segments, not considering segments 2, 5, 8, 11, and 17. Peak systolic strain was defined as minimal strain value of all time frames, while peak diastolic strain was characterized as minimal strain value of all time frames following early diastolic relaxation. Early diastolic circumferential strain rate (E_cc_/s) was used to measure early diastolic function and was calculated in 4 midventricular segments by dividing the change in strain between time frames by the temporal resolution (25 ms). E_cc_/s was defined as the slope from end-systole to mid-diastole as has been previously published [22].

### Statistics

Unless otherwise stated, data are presented as mean value ± standard deviation (SD). Data were statistically analysed by the paired or unpaired Student’s *t*-test. Regression analysis was done with Pearson product moment correlation. P values below 0.05 were assumed to be significant.

## Results

### Adjustment of GRASE for in vivo measurements of the human heart

In order to evaluate the effect of EPI on T2 values, we performed experiments in muscle-phantoms *ex vivo* and found that GRASE derived T2 values were in good agreement with T2 values of a standard MESE in repeated experiments (Additional file 1: Figure S1) by saving measurement time of factor 6. Figure 1A displays representative GRASE-derived images of a midventricular short axis slice in end-diastole at different echo times *in vivo*. Endocardial contours were drawn to illustrate the minor degree of cardiac movement during acquisition at end diastole. These images were used for fitting each voxel in the intensity echo time plot as shown below. For the assessment of optimal echo number, we calculated T2 maps with 5, 10 or 15 echoes with an interecho spacing of 10 ms. The T2 values derived from each voxel were calculated automatically by in-house developed software and plotted in a T2 value map as stated nearby. Here, T2 values are represented by a color code ranging from 0 ms (black) to 150 ms (red). Mean myocardial T2 values were calculated automatically after drawing the myocardial contours as region of interest (ROI, black). As given in the figure, mean T2 value within myocardial tissue raised dependent on the number of echoes used for calculation. 15 echoes with an interecho spacing of 10 ms were found to cover the complete exponential decay of intensity in myocardial tissue. As can be seen in Figure 1A, the endogenous myocardial movement in end-diastole between images at 10 and 150 ms was only very little. Besides that, strain rate analysis with SENC revealed that strain rate during data acquisition at end diastole was only 1.7 ± 2 Ecc/s. Thus, for the following measurements, we chose 15 echoes with an interecho spacing of 10 ms.

### Global myocardial T2 values in volunteers: inter- and intraobserver variability

**Figure 2:**
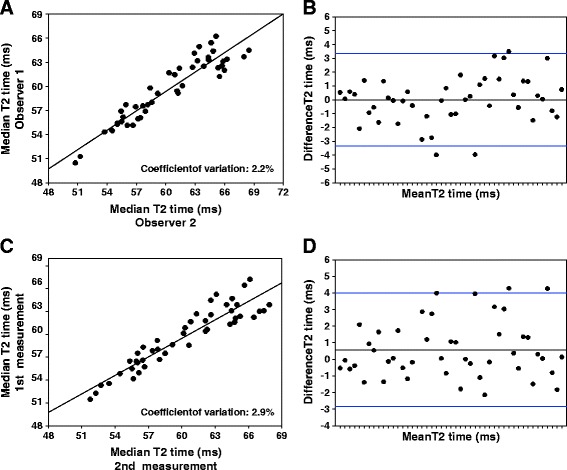
**GRASE-derived T2 values show low inter- and intra-observer variability. (A)** Interobserver variability for global median myocardial T2 values in volunteers for two experienced observers given in a scatter plot. The coefficient of variation was 2.2%. **(B)** Bland-Altman plot with limits of agreement in blue (1.96 × standard deviation). **(C)** Intra-observer variability for global median myocardial T2 values between 2 measurements. The coefficient of variation was 2.9%. **(D)** Bland-Altman plot with limits of agreement in blue (1.96 × standard deviation).

74 volunteers whose characteristics are given in Table 1 participated in the study and interpretable T2 maps could be generated in 97.5% of all cases. To investigate the variability of T2 values due to post processing, average T2 times of 45 volunteers were determined twice by two experienced observers. As can be seen in Figure 2A and 2B, there was a low inter-observer variability. The mean difference in T2 time was 0.004 ± 1.68 ms (R = 0.94), while the coefficient of variation was 2.2%. Low variance could also be documented for the intra-observer analysis (Figure 2C and 2D), where the mean difference of repeated measurements was 0.57 ± 1.7 ms (R = 0.91), with a coefficient of variation of 2.8%.

### Myocardial T2 values in volunteers: variations due to slice location, sex, and age

**Figure 3:**
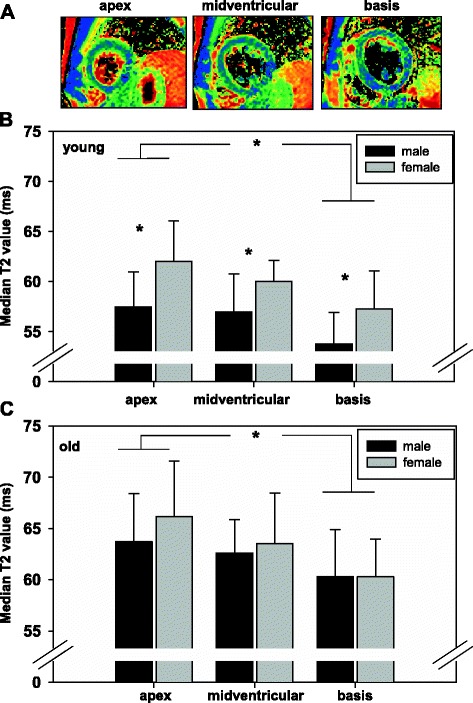
**Myocardial T2 values acquired** ***in vivo*** **are dependent on slice region, sex, and age in volunteers. (A)** Three myocardial short axis slices were acquired for analysis of median T2 time **(B)** Median T2 values of young volunteers as defined in Table 1 in apical, midventricular, and basal short axis slices. Male volunteers are indicated in black and female in grey. **(C)** The same analysis was performed for older volunteers as defined in Table 1. Values are given as mean ± SD. * = p < 0.01, male compared to female in the same short axis slice and between apical and basal short axis slices within the same sex.

Figure 3 demonstrates the results of the volunteer analysis. T2 maps were systematically acquired in 3 short axis slices as given exemplarily in Figure 3A (apex, midventricular and basis) and segmented according to the American Heart Association 17 segment model omitting segment 17. The results of younger volunteers are shown in Figure 3B. Here, median T2 values of apical short axis slices differed significantly from basal ones in both male (apical: 57.5 ± 3.5 ms; basal: 53.4 ± 3.1 ms, p < 0.01) and female volunteers (apical: 64.5 ± 4.0 ms; basal: 57.3 ± 3.8 ms, p < 0.01). Furthermore, female volunteers displayed significantly increased median T2 values in all short axis slices (p < 0.01 for each slice). Figure 3C displays the results of the older volunteers group. The slice dependent analysis revealed, that the apico-basal T2 gradient was also significant in the aged heart for males (apical: 63.7 ± 4.7 ms; basal: 60.3 ± 4.6 ms, p < 0.01) and females (apical: 66.1 ± 5.4 ms; basal: 60.3 ± 3.7 ms, p < 0.01), whereas the differences in myocardial T2 between male and female within the slices disappeared. The older volunteers group displayed significantly increased median T2 values compared to the respective sex and slice location in younger volunteers (all p < 0.01).

For regional T2 analysis, 1056 segments were included. Here (Additional file 1: Figure S4), posterolateral segments displayed significant decreased mean T2 values compared to anteroseptal segments. This was true for both genders, for each slice and in both age groups.

### Age-dependent difference in myocardial T2 values

**Figure 4:**
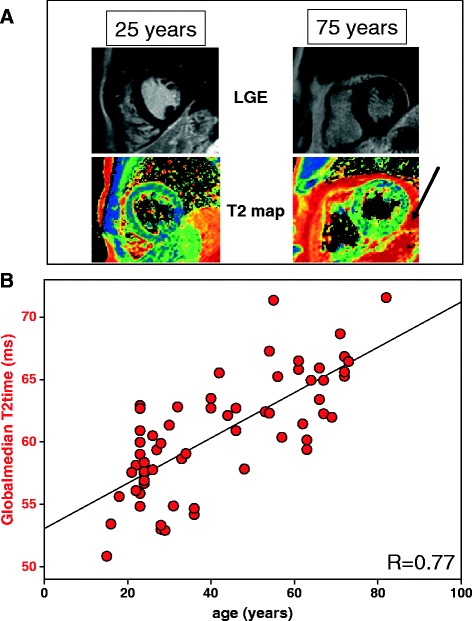
**Age correlates with global median T2 time in absence of known cardiovascular disease. (A)** Typical examples of two volunteers of different age (25 and 75 years) receiving T2 mapping and Late Gadolinium Enhancement (LGE). While there is no sign of a structural heart disease (LGE), T2 maps display differences in distribution of myocardial T2 values already upon visual inspection. Note the increased pericardial fat in the older volunteer (arrow). **(B)** Mean global T2 values increase with age (R = 0.77 with n = 69).

To further analyse the effects of aging on global myocardial T2 time, we correlated mean T2 time with age in our volunteers in Figure 4. Figure 4A exemplarily displays LGE and the corresponding T2 map of two volunteers of 25 and 75 years. While LGE displays no myocardial lesions, T2 maps already differ on visual inspection. Figure 4B shows a regression analysis of volunteers’ age and global myocardial T2 time, yielding a correlation with R = 0.77 (p < 0.01).

### Comparison of segmental and global T2 values with segmental and global strain as well as heart rate

**Figure 5:**
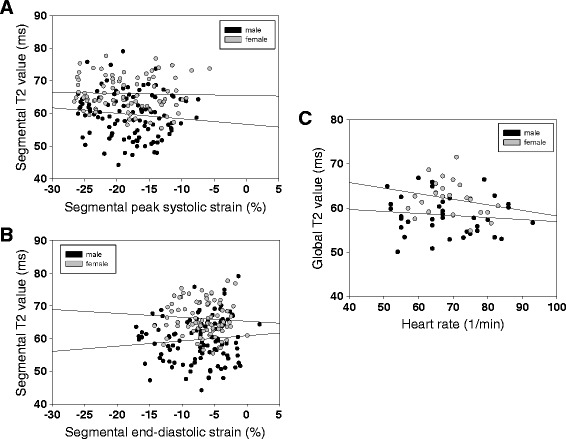
**GRASE-derived T2 values are not influenced by local myocardial strain or heart rate in volunteers.** Regression analysis of segmental T2 values and segmental strain in male (black) and female (grey) volunteers. No correlation was found between **(A)** peak systolic strain (%) or **(B)** peak diastolic strain (%) with mean T2 values (R < 0.1 for each sex and strain analysis). No correlation was also found between heart rate and global T2 time **(C)**.

To exclude an effect of wall movement on segmental T2 values, we acquired segmental circumferential strain values by SENC imaging of each myocardial segment analysed for T2 in 30 volunteers. In Figure 5 regression analysis of T2 values, peak systolic strain (Figure 5A) and peak diastolic strain (Figure 5B) for male (black) and female (grey) volunteers are presented. No correlation was found in any of these regressions (p > 0.3, R < 0.001) indicating that local maximal systolic or early diastolic strain had no effect on local T2 values. To analyse the effect of heart rate on T2 values, we performed a regression analysis of heart rate during data acquisition and T2 values (Figure 5C). First, we found no significant difference between the groups (HR: 69 ± 8 beats per minute (bpm) and 69 ± 11 bpm for young females and males; HR: 70 ± 5 bpm and 66 ± 11 bpm for old females and males). Secondly, there was neither a correlation between heart rate and myocardial T2 value across all volunteers (R = −0.1) nor in the respective subgroups (R = −0.26 and −0.13 for young females and males; R = −0.37 and −0.32 for old females and males, respectively).

**Figure 6:**
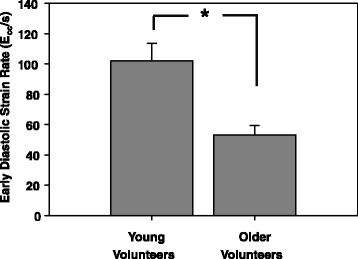
**Early diastolic strain rate is diminished in older volunteers.** Both groups of volunteers were analysed with respect to early diastolic strain rate (E_cc_/sec) which showed significantly diminished values in older volunteers. Values are mean standard deviation. * p > 0.01.

Since myocardial aging was shown to be characterized by impaired relaxation properties [21], we compared the early diastolic strain rate as measured by SENC imaging in both volunteer groups. Figure 6 shows, that older volunteers, formally characterized by increased T2 values, are characterized by a decrease in early diastolic strain rate as measured by SENC (Young: 101 ± 10.2 vs. Old: 53.12 ± 7.4 E_cc_/s, p < 0.05). However, myocardial strain rate during data acquisition was very low compared to early diastolic and systolic strain rate (1.7 ± 2 vs. 81.2 ± 20 vs. 45 ± 18 E_cc_/s, p < 0.01).

## Discussion

The present study demonstrates the feasibility of myocardial T2 quantification with GRASE in a large cohort of volunteers. Our results indicate that T2 values are quite comparable between individual hearts with respect to segment location and sex. Interestingly, aging of the human heart with representative cardiovascular risk factors is significantly correlated with increasing myocardial T2 values and paralleled by diastolic relaxation impairment. On a segmental basis, however, maximal systolic or diastolic strain as measured by SENC imaging is not correlated to segmental T2 values.

T2 mapping is a highly attractive technique for characterization of myocardial tissue in disease state, making use of T2 relaxation alterations accompanying ischemia or inflammation [7]. Thus, different vendors have recently placed several approaches for myocardial T2 mapping in humans on the market which differ in technical details. The widest used sequences are based on steady state free precession methods with varying T2 preparations for T2 mapping within one breath hold. Here, 3 preparation-times (0, 24, 55 ms) are used for calculation of local T2 times [11,14,15,23]. This has shown to be quite robust with good intra- and interobserver variability [14]. However, the complete coverage of exponential intensity decay of human muscle tissue provides the smallest T2 calculation error with more than 3 echoes [24]. As can be seen in Figure 1, calculated myocardial T2 values clearly depend on echo number and interecho spacing. For the present study, we chose 15 echos separated by 10 ms for the T2 value calculation. This set-up was chosen, to cover the whole exponential intensity decay of myocardial tissue along the acquisition of 15 echoes (Figure 1B, bottom). Importantly, the data acquisition along 150 ms in end-diastole was a compromise between least strain rate in end diastole (1.7 ± 2 Ecc/s) and maximal coverage of the myocardial intensity decay. Although mean myocardial T2 values with GRASE were higher compared to T2 prepared gradient echo sequences [11,14,23], they more closely mirror myocardial T2 values with increased echo image number [25,26]. Moreover, GRASE-derived T2 values were proven to be valid compared to a conventional MESE in muscle phantoms *ex vivo* while time saving by a factor of 6 (Additional file 1: Figure S1).

To validate acquisition and post-processing procedures, we performed several experiments in muscle phantoms. While GRASE was already investigated in the brain for the respective myelin-water environment [20], we showed that this sequence was also sensitive in the detection of global and local water changes in muscle phantoms *ex vivo* (Additional file 1: Figures S2 and S3).

Previous T2 mapping publications reported of quite high intersubject variability of myocardial T2 values in volunteers due to unknown co-factors [14,23]. Since stable cut-off values for differentiation of healthy and diseased myocardium are needed, this phenomenon accentuates the need for generation of reference values in a large group of volunteers. As can be seen from Table 1, we recruited 74 volunteers and separated them by age and sex. In line with previous reports, we observed a significant decrease in global myocardial T2 values from apex to base [23]. This effect was highly significant and more pronounced in male than in female volunteers. The reason has been debated to be most likely due to the partial volume effect rather than a morphological difference increasing towards the apex of the heart. The fact that we could observe this effect in volunteers irrespective of their age and sex makes this hypothesis most likely. Using reasonable slice thicknesses, this on the other hand means that these differences in T2 are unavoidable and therefore, a reference level has to be separately defined for all three cardiac regions (apex, mid-ventricle, base).

Furthermore, the finding of increased T2 values in anteroseptal segments compared to posterolateral ones was also in line with previous studies (Additional file 1: Figure S4) [23].

In contrast to previous studies, our analysis revealed significantly increased T2 values in female compared to male volunteers. This was true for all slices in younger volunteers, while it was only observed by trend in aged volunteers without reaching the level of significance. Previous studies presumed higher T2 value variability in females due to increased cardiac motion [14]. However, the results from our segmental strain and T2 value regression analysis revealed that T2 was independent of maximal systolic or diastolic strain. Additionally, the heart rate analysis showed that there were no significant differences in heart rates of the volunteers (Figure 5). It is well known, that gender-related androgens have a major impact on cardiac geometry, function and response to cardiovascular challenges [27]. Additionally, androgens lead to myocardial hypertrophy with impact on glycogen content and capillary density [28]. As expected, myocardial weight index was significantly higher in our male (74.3 ± 10.5 g/m^2^) compared to our female (55.0 ± 9.1 g/m^2^) volunteers (p < 0.05). While this finding would support increased T2 values in males compared to females as known for myocardial hypertrophy [8], we found the T2 time to be decreased in male volunteers. Thus, the direction of the T2 value difference (male < female) was somewhat surprising.

Most interestingly, global myocardial T2 values of our study were positively correlated with age (Figure 4). In line with this finding, the Baltimore and the Framingham studies have shown, that the aging myocardium displays features of ventricular hypertrophy and diastolic filling impairment [29]. While the myocardial weight index within our study did not significantly change between younger and aged volunteers, the diastolic filling pattern displayed significantly decreased early diastolic strain rates in the latter one (Figure 5). One explanation for this finding might be the increase in myocardial triglyceride content in aging hearts or the higher incidence of metabolic syndrome in the aged population [30]. Interestingly, increased triglyceride content was shown to be related to impaired diastolic filling patterns [31]. These features of the aging myocardium might parallel or be related to the increase in global T2. However, since we recruited older volunteers on the basis of a “real life” scenario, the typical prevalence of hypertension and diabetes might also explain the T2 value differences. The absence of hereditary cardiovascular diseases, normal electrocardiogram, normal indexed heart weight, normal cardiac stress testing (perfusion imaging) and absence of any LGE cannot exclude microvascular diseases or mild cardiac hypertrophy due to hypertension or diabetes hidden beyond the detection limits of those inclusion tests.

## Conclusion

This study provides evidence, that in a large group of volunteers global myocardial T2 values were increased in female volunteers compared to male without a correlation to local strain values. Besides that, age was correlated with increasing T2 values. According to previous findings of myocardial T2 quantification, we found an apico-basal and anteroseptal-posterolateral gradient of T2 time in myocardial tissue. Against the background of the present findings we propose, that T2 reference values needs to be created for each type of sequence. This has to be performed with respect to age and sex for exact discrimination between healthy and diseased myocardium.

## Additional file

Additional file 1:
**Online Data Supplement.**
