# Supplementary material for: Myocardial T2 mapping reveals age- and sex-related differences in volunteers
Source: J Cardiovasc Magn Reson. 2015 Feb 6;17(1):9. doi: 10.1186/s12968-015-0118-0 (PMC4318191; doi:10.1186/s12968-015-0118-0)
Supplement: Additional file 1: — Online Data Supplement. [file 12968_2015_118_MOESM1_ESM.pdf]

Online Data Supplement

Online Data Results

A phantom study with commercially available cow meat was conducted to validate GRASE against a conventional multi echo spin echo (MESE) sequence (TR = 1500 ms, TE = 15 x 10 ms, FA = 90°, res: 10 x 2 x 2 mm^3). GRASE parameters were standardized for validation (TR 1500 ms, TE 15 x 10ms, FA 90°, res 10 x 2 x 2 mm^2). As can be seen in supplemental figure S1, GRASE derived T2 values in cow meat were not significantly different from values acquired with the MESE (GRASE: 54.0±5.0 ms; MESE: 51.4±5.3 ms; p=0.45 with n=4 experiments). Compared to MESE, GRASE derived T2 maps were acquired 6 times faster (MESE: 61 sec, GRASE: 11s acquisition time).

For sensitivity evaluation of GRASE-derived T2 values in terms of detecting changes in tissue water content, we conducted experiments with varying global and local tissue water in muscle phantoms. Figure S2 highlights the result of an experimental series with cow meat of decreasing tissue water content as a result of drying by vacuum heat. As can be seen, T2 values are decreasing with decreasing water content.

To prove that the effect of decreasing T2 values in response to drying is reversible, we conducted experiments with meat exposed to dehydration by osmotic forces (salting) and rehydration. Figure S2A displays that salting exposure (180 min) leads to decrease in mean T2 time (fresh: 56.61±1.8 ms; salting: 48.19±1.2 ms, $p<0.05$), while rehydration for 30min increases mean T2 value (salting: 48.19±1.2 ms; rehydration: 52.42±1.3 ms, $p>0.05$) significantly. To investigate whether the observed effect of global tissue water change on mean T2 value could also be reproduced for local changes (Figure S2B) we injected different amounts of oil (1=500 µl, 2=1 ml) and NaCl (3=500 µl, 4=1 ml) into a meat phantom. After injection, there were no visible changes in tissue appearance upon macroscopically inspection. Fat, as well as water injection led to an increase in local tissue T2 time. Equivalent volume of

water led to a larger increase in local T2 time as compared to fat (1: 66.75 ms vs 3: 77.44 ms and 2: 69.59 ms vs. 4: 82.56 ms).

Supplemental Figures

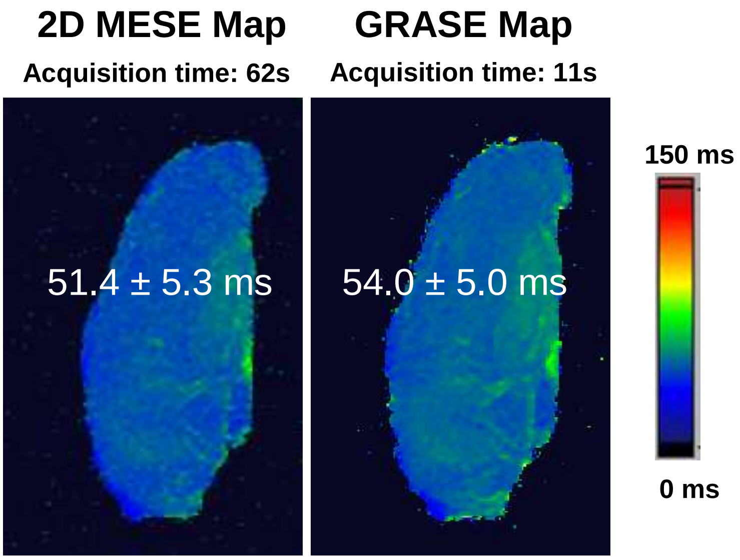

Figure S1

Validation of gradient and spin echo (GRASE)-derived T2 values against a multi-echo spin-echo (MESE) sequence in cow meat. Acquisition time was 62 seconds with MESE and 11 seconds with GRASE. T2 value calculation is given in the figures with mean ± standard deviation. T2 values are given in a color-coded map assigning 0 ms to black and 150 ms to red as indicated in the colour bar.

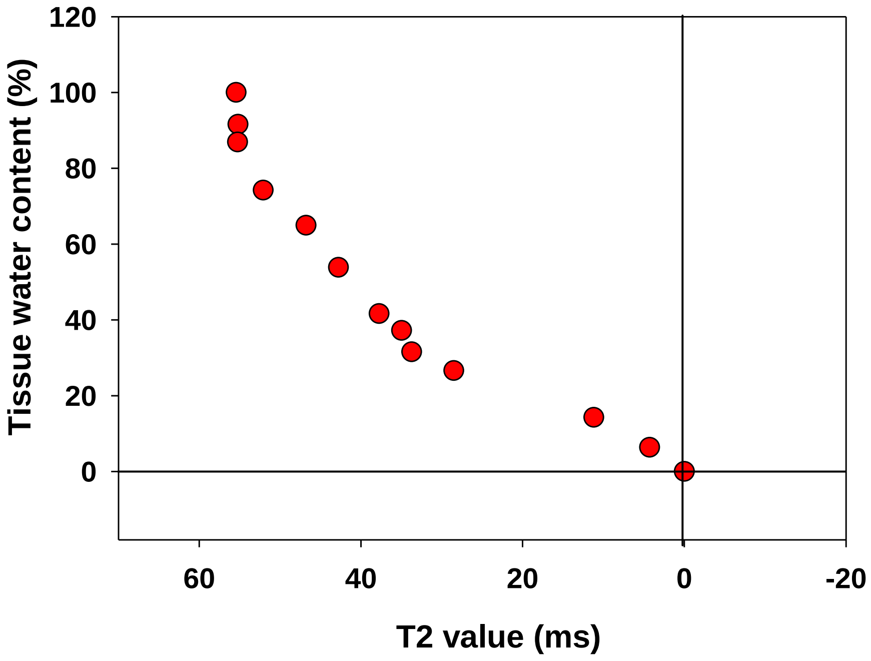

Figure S2. Tissue water content determines T2 time in muscle phantoms as measured with GRASE

Meat phantoms were treated by vacuum concentration for reduction of tissue water content given in %. T2 value calculation was done with GRASE.

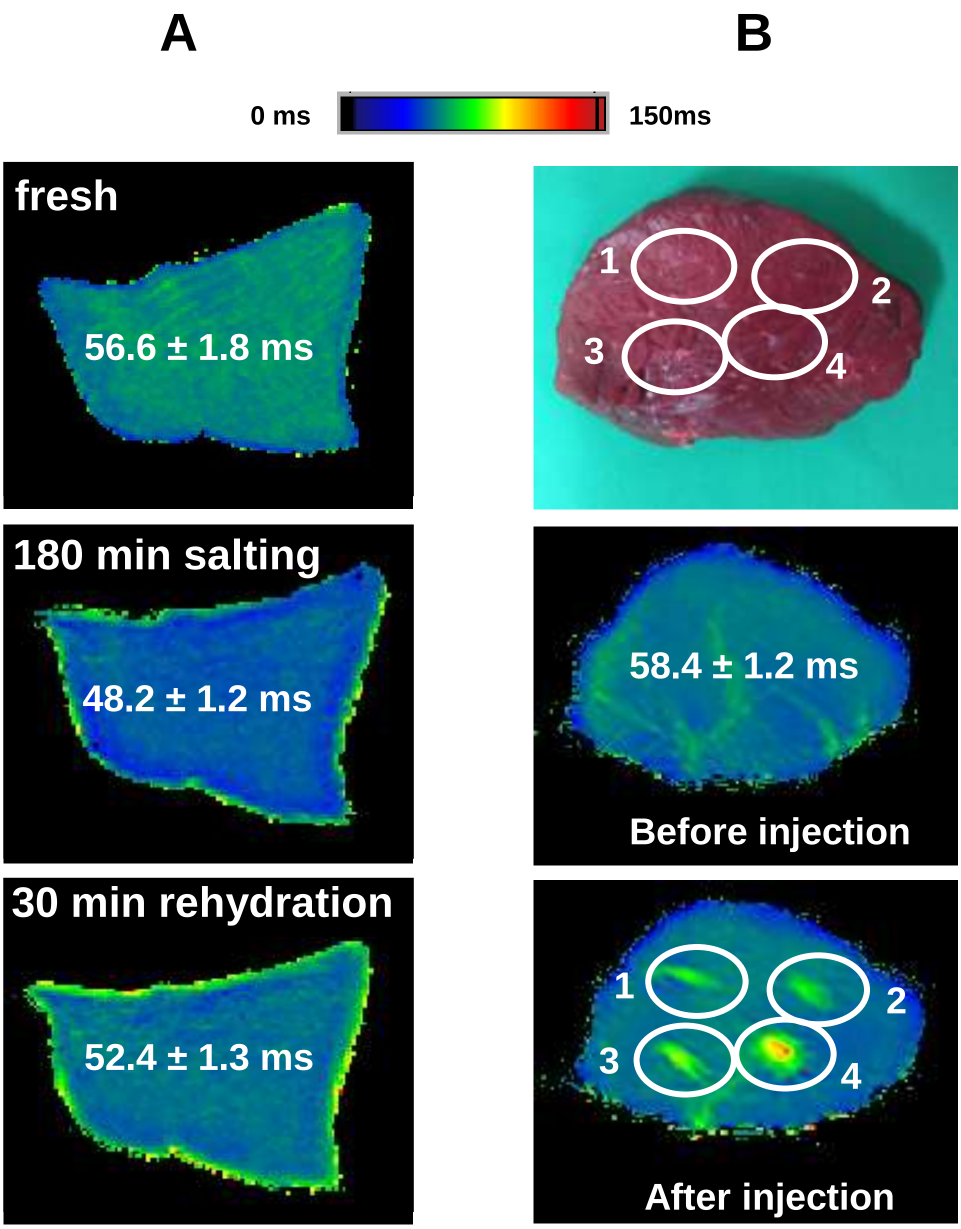

Figure S3

Sensitivity of GRASE in detecting changes in global and local tissue water. **(A)** Fresh cow meat (top) treated with 180 minutes of salting (middle) and 30 minutes of rehydration (bottom). Mean T2 value decreased by 14% from top to middle image before returning to baseline values in bottom image. **(B)** Focal injection of different volumes of oil (injection sides 1 = 500 µl + 2 = 1000 µl) and water (injection side 3 = 500 µl + 4 = 1000 µl). While there was no visible change in native tissue after injection (top) there was a marked increase in local T2 time after injection (compare middle and bottom image). T2 values are given in a colour coded map, assigning 0 ms to black and 150 ms to red.

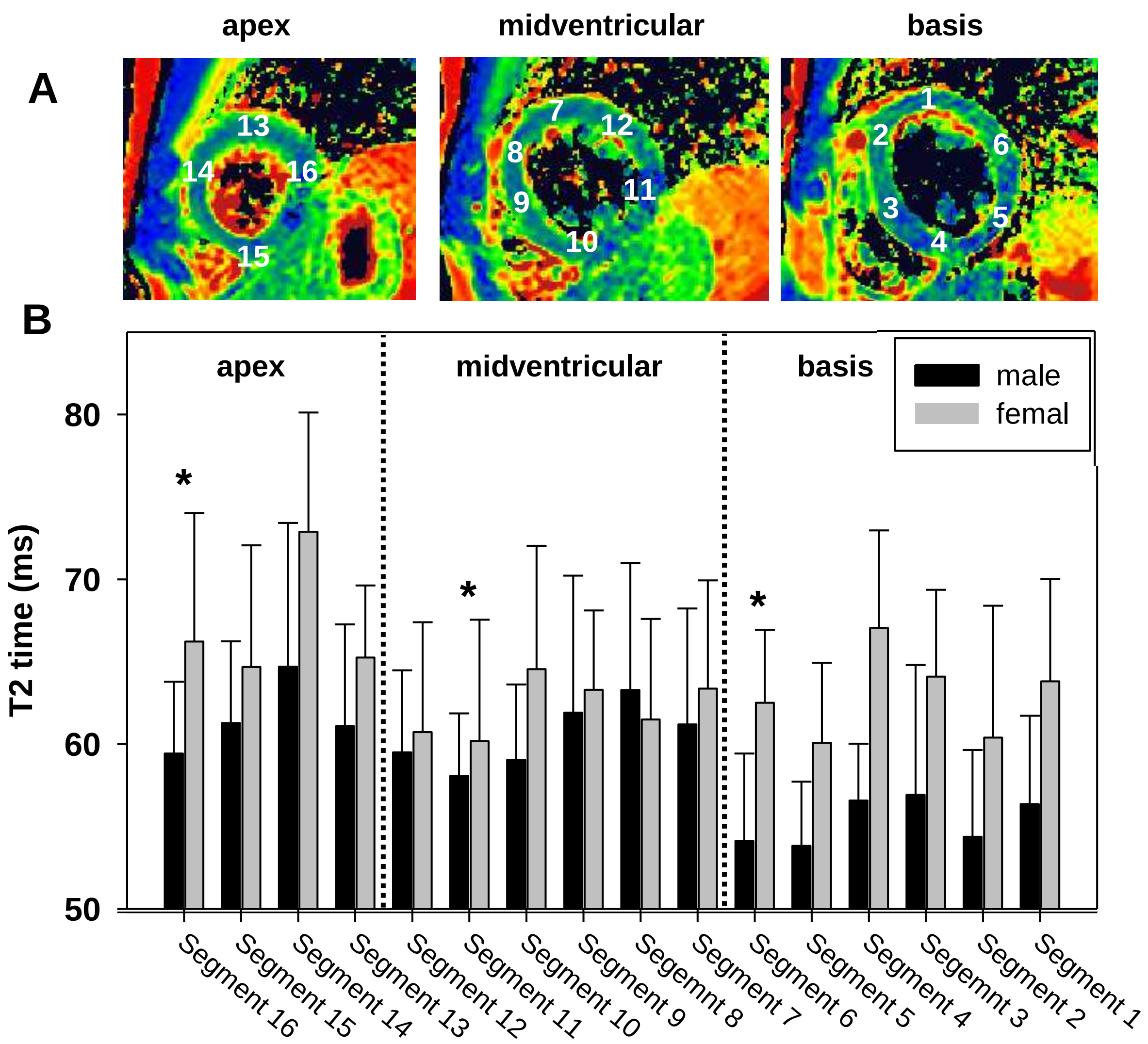

Figure S4

Segmental analysis of myocardial T2 values according to the 17 segment model omitting segment 17. (A) Representative three short axis slices of apex, midventricular and basis with imprinted segments. (B) Analysis of 1056 segments. Values are given as mean ± SD. Segment 16 against 14, segment 11 against 8 and segment 5 against segment 3: *<p 0.05
